# Effect of Physiological Saline Solution Contamination on Selected Mechanical Properties of Seasoned Acrylic Bone Cements of Medium and High Viscosity

**DOI:** 10.3390/ma14010110

**Published:** 2020-12-29

**Authors:** Robert Karpiński, Jakub Szabelski, Przemysław Krakowski, Józef Jonak

**Affiliations:** 1 Department of Machine Design and Mechatronics, Faculty of Mechanical Engineering, Lublin University of Technology, Nadbystrzycka 36, 20-618 Lublin, Poland; j.jonak@pollub.pl; 2 Section of Biomedical Engineering, Department of Computerization and Production Robotization, Faculty of Mechanical Engineering, Lublin University of Technology, Nadbystrzycka 36, 20-618 Lublin, Poland; 3 Chair and Department of Traumatology and Emergency Medicine, Medical University of Lublin, Staszica 11, 20-081 Lublin, Poland; przemyslaw.krakowski84@gmail.com

**Keywords:** bone cement, contamination, saline, mechanical parameters, compressive strength, microhardness, seasoning, statistical analysis

## Abstract

Bone cements play a key role in present-day surgery, including the implantation of hip and knee joint endoprostheses. The correct and durable bonding of the prosthesis to the bone is affected by both the static strength characteristics determined in accordance with ISO 5833:2002 and the resistance to long-term exposure to an aggressive environment of the human body and the impurities that may be introduced into the cement during implementation. The study attempts to demonstrate statistically significant degradation of cement as a result of the seasoning of cement samples in Ringer’s solution with simultaneous contamination of the material with saline solution, which is usually present in the surgical field (e.g., during the fixing of endoprostheses). The results of statistical analysis showed the nature of changes in compressive strength and microhardness due to seasoning time and degree of contamination.

## 1. Introduction

Bone cements introduced by Charnley [1,2] are currently among the most commonly used polymer composites in orthopaedics and traumatology [3]. In surgery, these materials are mainly used to fix joint endoprostheses in hip and knee alloplasty [4,5] and to fill in bone defects in order to make up for the lack of bone tissue and strengthen internal stability in pathological fractures [6] and, for example, during minimally invasive vertebroplasty or kyphoplasty procedures performed with compression fractures [7,8,9,10]. They are also used in other cases of reconstructive surgery procedures. However, they are most often used as binding material for arthroplasty procedures. More than one million hip arthroplasty procedures alone are performed worldwide each year [11]. It is a procedure that enables the restoration of painless joint function in the final phase of degenerative changes. It is estimated that 40% of men and 47% of women may be affected during their lifetime [12]. The risk of developing degenerative changes increases with the age of patients; however, a number of publications indicates the occurrence of degenerative changes in younger people as well [13]. Large joint arthroplasty, which is a procedure with an established position in the orthopaedic world, is unfortunately exposed to numerous complications forcing the revision arthroplasty to be performed. For example, the fifteen-year lifetime of knee prostheses is estimated at 90% [14]. In the case of hip joint prosthesis, the average age of a patient undergoing revision arthroplasty is 71.4 years and that of the knee joint—68.2 [15]. With an estimated increase of up to 600% in the number of large joint treatments performed until 2030 [16], it can be assumed that more and more young people will have to undergo a revision procedure. However, the results of revision procedures differ significantly from the results obtained in primary arthroplasty procedure [14].

The vast majority of revision procedures is caused by aseptic loosening of the prosthesis [17], which may be affected by the quality of bonding of the prosthesis elements to the bone with bone cement. The prosthesis cementation process itself has no clear guidelines to the present day [18].

When analysing the basic applications of bone cements, it can be assumed that the basic factors determining the biofunctionality of these materials are: The ability to carry static and dynamic loads, enabling reliable operation of the joint even under extreme loads, the ability to dampen vibrations and adequate absorption of overload, resistance to frictional wear and tear, biotolerance and stimulation to bone mass increase/decrease [19,20,21]. The most important mechanical properties of cement include the compressive, tensile, shear and fracture strength. The differences in these properties are related to differences in composition, mixing methods, ageing, temperature and viscosity during application [22,23,24,25,26,27,28]. Currently, many types of bone cements are used in the form of pure polymer or with structural admixtures of various substances to improve physical properties. The use of fillers has a significant impact on mechanical properties [5,29,30].

After implantation, bone cement works in a very aggressive environment, which increases the rate of its ageing, and this in turn causes a greater risk of cement flaking, as a result of which it weakens the prosthesis–centre–bone bonding. This phenomenon may lead to loosening of the endoprosthesis and the need for a revision procedure and another implantation [5,29,31]. In cement-based arthroplasty, cement is a component that defines the durability of the prosthesis-cement and cement-bone bonding. There are many hypotheses saying that aseptic loosening of the implant is preceded by destruction of bone cement [32,33,34,35]. Although bone cements based on polymer composites are widely used, they do not guarantee mechanically and biologically stable bonding to the bone and, above all, are susceptible to bacterial adhesion and infection development [36]. It is estimated that more than 25% of prosthetic implants may indicate signs of aseptic loosening [37,38,39].

Bearing in mind the above, an attempt was made to determine the influence of the degree of contamination of bone cements, including seasoning in saline solution, on selected mechanical parameters of two bone cements of different composition, their application and physical characteristics.

## 2. Materials and Methods

### 2.1. Materials

The materials selected for testing are commercially available cements:DePuy CMW 1 (DePuy Synthes, 325 Paramount Drive, Raynham, MA, USA) high viscosity medium-setting time bone cement and DePuy CMW 3 (DePuy Synthes, 325 Paramount Drive, Raynham, MA, USA) medium viscosity bone cement with a medium setting time. Each of the cements selected for testing is supplied as a two-part product containing a separately sterile liquid component and powder, which must be mixed before use to form a finished cement. Polymethyl methacrylate (PMMA) is a polymer belonging to the category of acrylic resins. It is obtained from a mixture of a monomer (methyl methacrylate, MMA) and polymer (particles of pre-polymerised methyl methacrylate) in the presence of an initiator, activator and stabiliser [36,40]. The liquid component of bone cement is a colourless, flammable liquid with a characteristic smell, whose main ingredient is methyl methacrylate monomer. Hydroquinone, which is a component of the liquid component, acts as a stabilizer to prevent premature polymerization, which can occur when exposed to heat or light. *N*,*N*-dimethyl-p-toluidine, on the other hand, is the component that triggers the polymerization process after mixing both components. Bone cement powder is white in colour and consists of a well-comminuted polymer based on polymethyl methacrylate. The benzoyl peroxide contained in the powder is responsible for initiation of polymerization after mixing the powder with the liquid. Detailed compositions of test materials expressed in % w/w are shown in Table 1.

### 2.2. Sample Preparation

The compression test specimens were prepared in accordance with international standard ISO 5833:2002 [41] Implants for surgery—acrylic resin cements, and were cylindrical in shape with a diameter of 6 ± 0.1 mm and a height of 12 ± 0.1 mm. The samples were made with an extra height and then ground to the described required size using abrasive paper with an average grain size of 35 µm. The diameter of the samples was uniform throughout the whole height and the bases were parallel to each other and perpendicular to the longitudinal axis of the sample. Prior to testing, the samples were subjected to a visual quality control to reject those with visible structural defects. In order to extend the polymerization time, both the liquid component and the powder were cooled to 16 °C; this procedure allowed for more precise filling of the mould. A 0.9% isotonic saline solution in the amount of 1, 3, 6, and 10% w/w, respectively, was introduced at the component pooling stage. Uncontaminated samples were also inspected. The samples prepared in this way were subjected to a process of seasoning in saline solution for 1, 15 and 30 days, respectively. Samples that were air-seasoned for 24 h were also tested. Batches prepared in this way, consisting of a minimum of 6 samples, were subjected to compressive strength, microhardness and weighing tests to estimate the weight gain due to liquid absorption.

### 2.3. Mechanical Testing

Compressive strength tests were conducted on the testing machine MTS Bionix–Servohydraulic Test System with dedicated MTS TestWorks software (Eden Prairie, MN, USA). The velocity of the material compression has been determined in accordance with ISO 5833:2002 [41] at 20 mm/min. As recommended in the standard, the number of samples tested in each batch should be at least 5, and in the case of tests, the number of samples tested should be between 6 and 8 per batch.

### 2.4. Statistical Analysis

A statistical analysis to determine whether there are statistically significant differences between the tested values of mean compressive strength of individual batches of medium- and high-viscosity bone cements, doped with and seasoned in saline solution, was carried out using Statistica 13.1 (Tulsa, OK, USA). The level of statistical significance in the study was adopted at α = 0.05.

Tukey’s HSD (honestly significant difference) test was used to for the multiple comparison of several averaged groups and to separate homogeneous groups with statistically insignificant differences. It is one of the few available post-hoc tests, and the only one that allows for testing groups of unequal numbers, which was the nature of the results of the analysed strength Due to the rejection of damaged samples, their number differs across the groups. Other multiple comparison test methods available in Statistica software are: Scheffé’s method, Newman–Keuls method, Duncan’s test and Fisher’s LSD (least significant difference) test. They also differ in the degree to which they obtain statistically significant results, as separated into the so-called liberal and conservative ones. “Conservative tests” are those where it is more difficult to obtain a statistically significant result and “liberal” ones are those where it is easier to obtain significant differences in averages [42].

## 3. Results

### 3.1. Change in Strength over Time, Depending on the Degree of Contamination

The results of the experimental compressive strength tests are presented in Table 2. The table shows for each batch the mean value (MV) of compressive strength, standard deviation (SD) and percentage coefficient of variation (COV) obtained in the series of results.

The high homogeneity of the results obtained is clearly visible. Statistical knowledge and sources define the interpretation of COV < 20–25% as low variability. The variability rate of average results within a batch of individual samples, in all batches, except one (CMW 1, 1%, 30 days), reached values of about 7% on average (not more than 11.4%). This shows low variation of the measured characteristic and at the same time indicates precise measurements. It is difficult to find specific relations between the variable parameters in the study and homogeneity results. Sometimes samples subjected to longer seasoning time were observed to be characterized by higher scatter of results within a batch than those seasoned for a shorter time. It is impossible to notice a similar behaviour with a change of quantitative contamination in the composition of the cement.

**Figure 1:**
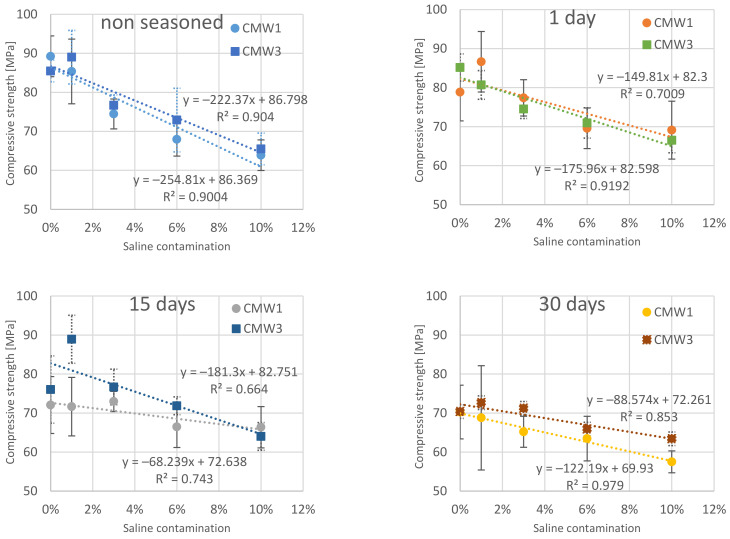
Dependence of cement compressive strength on the degree of contamination in groups according to the time of seasoning in the saline solution.

**Figure 2:**
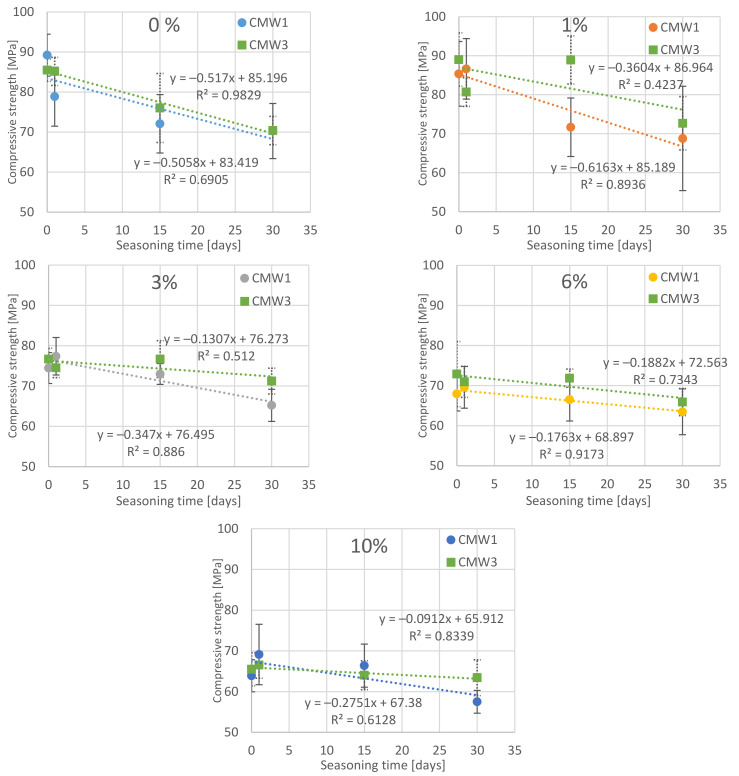
Dependence of cement compressive strength on the time of seasoning in the saline solution in groups according to the degree of contamination with saline.

The obtained values of compressive strength should be analysed according to the values of two variable test parameters for each cement: degree of contamination and seasoning time, and the nature of their variability. The results obtained, summarised twice in different grouping configurations, are presented in Figure 1 and Figure 2. In the first case, the results of experimental tests were grouped according to the seasoning time and the change of the examined feature was presented along with an increase in the degree of cement contamination. In the second case, the results of samples with the same method of preparation (degree of contamination) were grouped and the effect of seasoning time on compressive strength was analysed.

In order to show the trend of changes, linear trend lines were determined, for each summary, which assigned the value of the time variable determining the seasoning time or the quantitative degree of cement contamination to the average values of the experimentally obtained cement compressive strength.

The tables below present the results obtained (i.e., distinctions of homogeneous groups), statistically not differing from each other (A, B, C) at α = 0.05, as well as the results of analysis of significant differences between individual batches. The results are presented twice, depending on the way the dependent parameters are grouped, in Table 3 and Table 4, respectively.

The results of the statistical analysis explain the uncertainties associated with the differences between the individual batches more clearly. Determined were groups which constitute homogeneous sets of results which do not differ from each other at the assumed materiality threshold α = 0.05. One can observe variation in strength within the assumed time frame. In many cases, the results obtained on days 0, 1, 15 were not significantly different. A similar situation was sometimes found in the summary of results from days 15 and 30. In rare cases, especially with significant degree of pollution, statistically significant differences were not recorded in the whole time frame of the test. It has been confirmed that contaminated cements do not degrade as much with seasoning time. Their strength, usually already significantly lower than that of cements with a low degree of contamination, does not deteriorate to such a high degree. No more than two groups of homogeneous results were recorded for samples grouped with the same degree of contamination. A greater variability was observed by analysing the results of statistical analysis in the case of grouping the results according to the same seasoning time in the saline solution environment. Sometimes, even three homogeneous groups were recorded within the examined scope of variability of the degree of cement contamination (0%, 1%, 3%, 6%, 10%), especially for the initial seasoning times. The longer the time the cement stays in the solution, the stronger the impact of the contamination on the strength. For example, a comparison of the absolute differences between the average strength of the least and most contaminated batches of cement samples, between the unseasoned and the 30-day seasoned samples, shows that the decrease in strength with the degree of contamination was in this comparison twice as high for the 30-day samples as for the unseasoned ones. This confirms previous observations.

**Figure 3:**
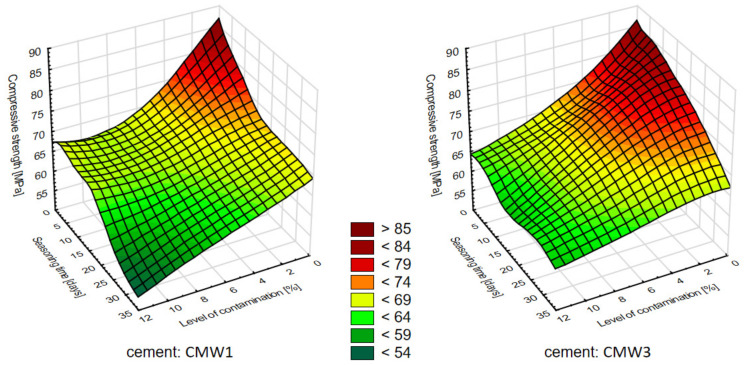
Surface diagram showing the dependence of the compressive strength on the seasoning time and amount of contamination.

**Figure 4:**
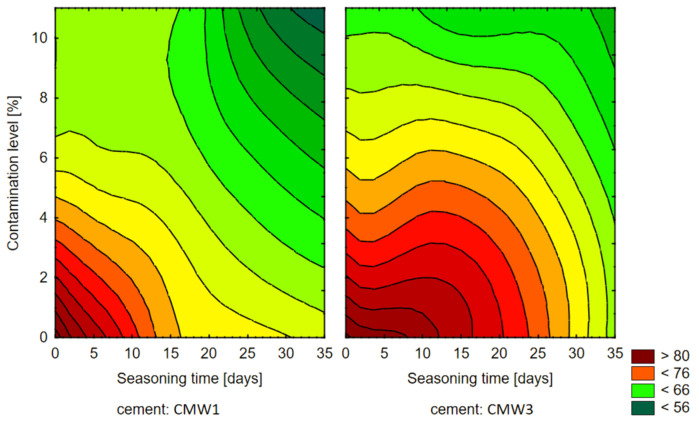
Contour diagram showing the dependence of compressive strength on seasoning time and amount of contamination.

Knowing the results of the influence of the analysed variable parameters of the degree of contamination and seasoning time on the compressive strength of the cement samples, an attempt was made to compile a summary of this influence in the form of the plane—of a 3D diagram. Figure 3 presents the planes generated for the average strength values of both cements. Figure 4 shows contour diagrams, which are projections of 3D diagrams from above while combining points of equal strength and generating homogeneous areas from them.

### 3.2. Change in Microhardness over Time, Depending on the Degree of Contamination

**Figure 5:**
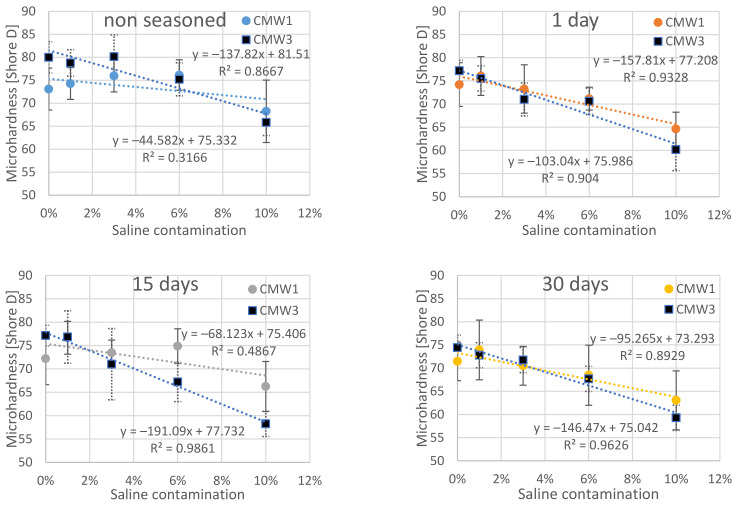
Dependence of cement microhardness on the degree of contamination in groups according to the time of seasoning in the saline solution.

**Figure 6:**
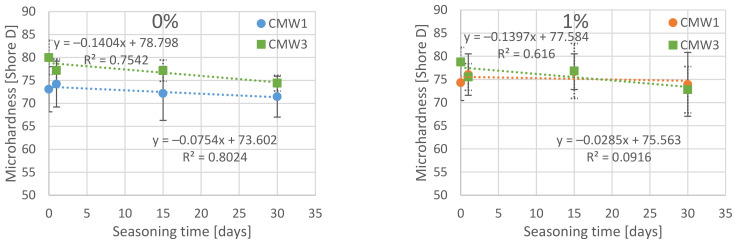

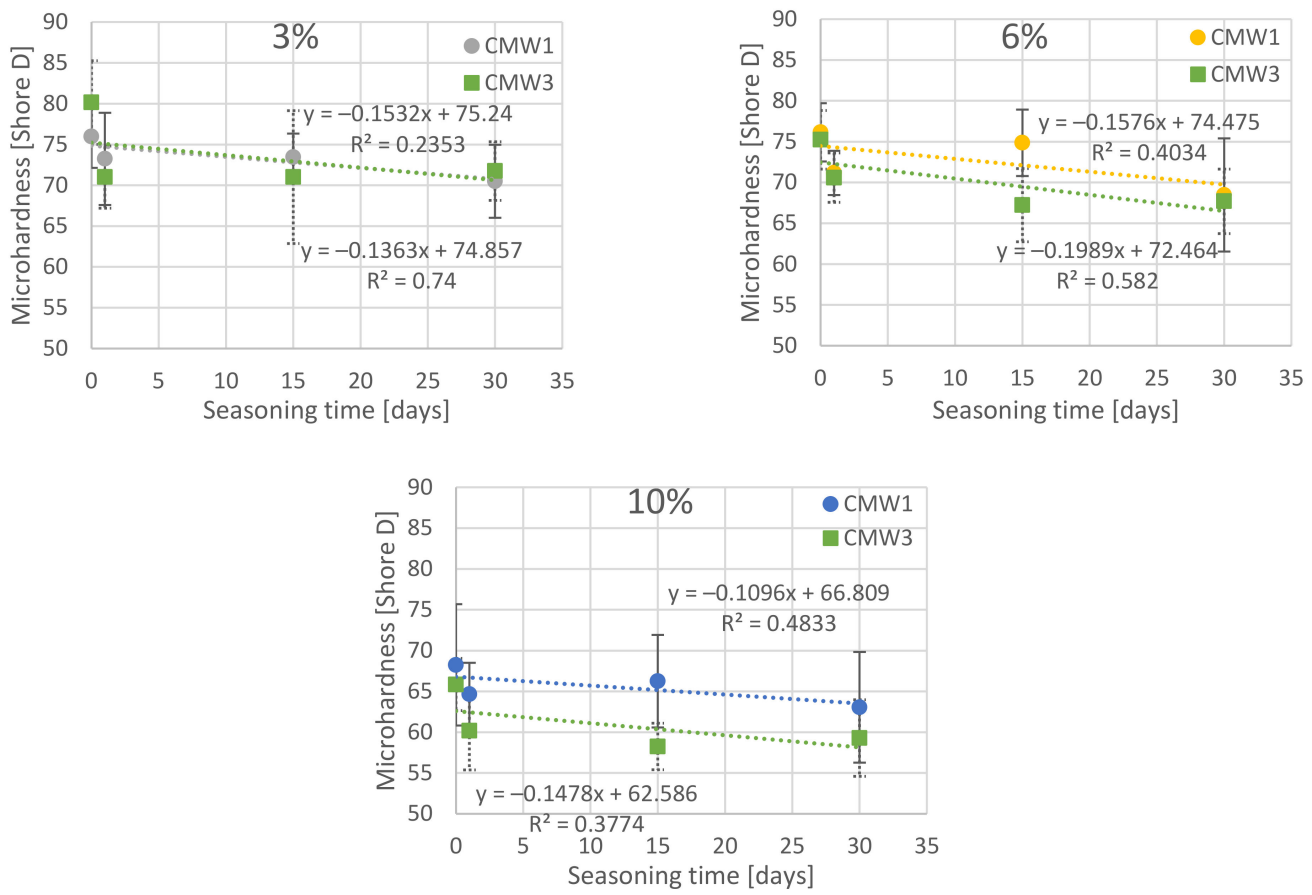
Dependence of cement microhardness on the time of seasoning in the saline solution in groups according to the degree of contamination with saline.

The results of the measurements of microhardness of the surface of the tested cements carried out over a period of 30 days are presented in a similar way as the above described results of compressive strength in Table 5. Figure 5, in turn, presents a summary in which the results or subsequent test days are grouped together and the change in hardness is shown depending on the degree of contamination changes. Results for the opposite configuration are summarised in Figure 6.

Confirmation of observation results should be sought in the results of statistical analysis. The following tables summarise the overall results of Tukey’s statistical tests, which are described in more detail in the chapter on statistical analysis of compressive strength. As before, statistically homogeneous subgroups have been distinguished within the groups. They were compared with the results of the analysis of significant differences between particular sub-batches. The results are presented twice, depending on the grouping of dependent parameters (degree of contamination and seasoning times) in Table 6 and Table 7, respectively.

On the basis of detailed analysis of the obtained results, it can be concluded that statistically significant degradation of microhardness across the seasoning time was recorded for CMW 1 basically only when the samples remained in saline solution for 30 days, and not for all batches (only the one contaminated in 6%). In the case of CMW 3, a significant decrease in microhardness was confirmed for almost all cases of contamination, except 1%. In the remaining variants of pollution, statistically significant differences (degradation) were sometimes recorded even as early as after 15 days. Analysis of the results in groups with the same seasoning time makes no room for any general conclusions. CMW 1 cement most often shows no significant changes in microhardness in the range 0–6%; only sometimes, at 10% contamination, the microhardness reaches values significantly different (smaller) than those of cleaner cements (samples seasoned for 15 days). The second material under study, CMW 3, is much less durable and statistically significant changes of weakening character are much more visible. For the unseasoned cement, weakness is recorded only at 10% contamination, but with longer seasoning time it becomes more and more visible, with 6% and 3% contamination, respectively, for 30 and 15 days of seasoning.

**Figure 7:**
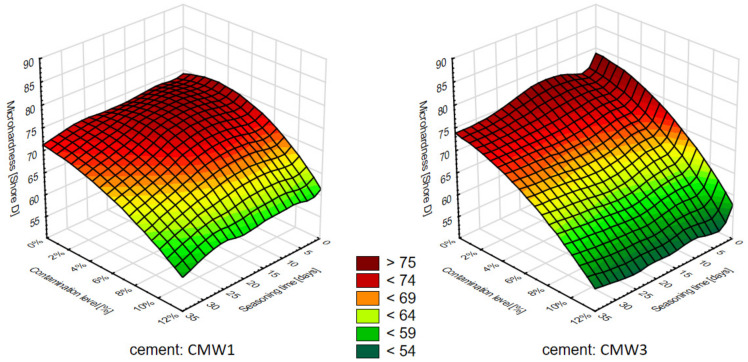
Surface diagram showing the dependence of microhardness on the seasoning time and amount of contamination.

**Figure 8:**
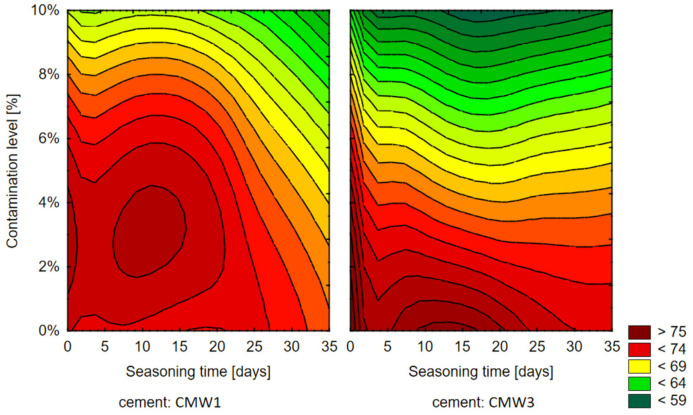
Contour diagram showing the dependence of microhardness on seasoning time and amount of contamination.

Based on the above results of the influence of the analysed variable parameters of pollution degree and seasoning time, 3D surface diagrams were prepared, corresponding to those for compressive strength (Figure 7), followed by contour diagrams (Figure 8)—homogeneous areas in the 3D chart, view from above.

## 4. Discussion

The durability of a fixing element depends on many factors. One of the most important elements is the correct fixing of the endoprosthesis using bone cement in the bone canal and the appropriate strength parameters of the cement itself. After the implantation, it is subjected to loads resulting from the patient’s movement, as well as to the aggressive environment in which it is placed. This is due to typical contaminants encountered during the surgery, such as saline, used to clean the marrow canal and to cool the cement immediately after implantation to reduce the risk of damage to surrounding tissues, as well as natural blood or small bone fragments that may affect the strength parameters of the material (degrade it over time—by default).

Each joint prosthesis has a specific survival time, after which a revision procedure has to be carried out. The most common cause for late revisions is aseptic loosening of prosthesis components [17,43]. Moreover, the number of treatments is growing year by year, whereas, according to the data of the Swedish Knee Arthroplasty Register (*Swedish.* SKAR—Svenska knäprotesregistret), the number of performed knee arthroplasty procedures has increased by 17% over four years and more than 90% of them are fixed with bone cement [44]. It is estimated that the fifteen-year lifetime of cemented knee replacements is only 90% [45]. Therefore, it is exceptionally important to assess the quality of bonding of the prosthesis with bone using bone cement. It should also be noted that the vast majority of large joint revision arthroplasty procedures are performed using bone cement. Furthermore, cement-based revision arthroplasty is more prone to aseptic prosthesis loosening than cement-free revision procedure [46]. This may be affected by contaminants entering the bone cement during its preparation.

The total strength of bone cement depends on many factors related to the way it is prepared, the mixing process, the use of intentional admixtures to improve mechanical parameters, and also the presence of the above mentioned contaminants introduced during its implantation, such as blood or bone tissue residues [22,24,39].

The mere attempts to modify the composition of bone cements are usually associated with the need to improve its selected properties. For example, the influence of mixing bone cements with graphene powder and its oxide was analysed. As little as 0.1% w/w had a positive effect on the, both static and fatigue, strength characteristics of the material. It also revealed the presence of functional groups on the surface of graphene oxide (GO) powder, which improved dispersion and facilitated strong chemical bonds between the filler and cement [47,48].

The degree of admixture is qualified in case of antibiotics admixture as small for up to 5% w/w and as large dose at the amount > 5% w/w. In the case of high doses, the possible risk of changes in the characteristics of the cement itself, including its strength, has been confirmed [36]. Nevertheless, organic fillers, which perform antibacterial functions, were tested. A 7% addition of encapsulated paraben nanoparticles did not degrade the cement in terms of compressive strength [49], and the same result was observed for antibacterial quaternary amine monomer (QAMA) [50].

Analyses related to the presence of saline in the cement composition are also known. The admixture of this material was studied, among others, to a large extent at 10–30% [51]. Although saline often occurs in surgical practice in the bone cavity during surgery, this amount cannot be treated as resulting from accidental contamination. However, the aim of the research was to check whether less stiff cement could be obtained, which would be better for osteoporotic spongy bone (equalization of stiffness). One of the observations was a slight decrease in polymerization temperature with the concurrent shortening of the curing time. No chemical effects of the admixed material on the tested cement were recorded. The change of the above mentioned properties was mainly caused by the observed increase in porosity (i.e., physical factors). However, the above-mentioned research did not take into account the degradation of cement over time.

Studies on admixture were mostly carried out using calcium hydrogen phosphate dihydrate (bruscite) in the wide range of 0–50%, adding it to the cement in two ways: as composite bone additives (CBA) (leaving all other components the same) or as composite bone substitutes (CBS) (removing dry polymer PMMA). Statistically significant degradation of compressive strength was not recorded up to the threshold of 10% admixture. A regular decrease in compressive strength was recorded from 18% upwards [6].

The environment, which is aggressive to cement, in which the prosthesis is cemented during the procedure, and the time during which the prosthesis/cement is used under these conditions, also have an adverse impact in this respect. Attempts have been made to simulate the above mentioned conditions during the cement tests, by seasoning them in Ringer’s solution, among others, or in temperature increased to values close to human body temperature. Many tests have shown deterioration of mechanical properties of cement due to the fact of seasoning in water, Ringer’s solution or plasma and the increasing degree of degradation over time. The time horizon of the tests was very extensive: From hours from cement polymerization reaction to years of seasoning [23,52,53].

There were also voices of criticism concerning the imperfection of the standards defining the scope of testing strength characteristics of cements [54], including those that address the problem of disregarding either the working conditions of the cement or its seasoning time [55].

Combining the above mentioned problems, research was undertaken to determine the dependence of the degree of contamination of cement with saline solution on selected strength parameters in the situation of cement seasoning in an in vivo simulation environment. The relatively large range of contamination degree, adopted for the study, results not so much from the estimated quantity of admixture that can occur clinically, but more from the attempt to indicate a specific degree of contamination for which the material will no longer meet the initial strength specified in the standard.

The first observations of the obtained results allow us to see the preliminary relations between the variable parameters of the tested cements and their final compressive strength values. First of all, there is a clear change (decrease) in average compressive strength with seasoning time under conditions simulating in vivo environment. Whether we are looking at CMW 1 or CMW 3 cement, the trend line always indicates a deterioration in strength over time, although it can be seen that for CMW 1 the decrease is greater (the straight line is more often inclined downwards) than for CMW 3. The degradation in cement seasoning is also related to the state of contamination. The graphs of average values indicate that the higher the percentage of saline admixture, the lower the strength of the cement just after its formation (and curing), but at the same time the degree of strength decrease with seasoning time appears lower. The trend line directional coefficients that describe the slope angle of the trend line become smaller in absolute terms as the cement becomes cleaner. Interestingly, after just two to four weeks of seasoning, the strength of samples of both cements falls below the minimum threshold of 70 MPa as per ISO 5833:2002 [41]. This value, of course, applies to hardened but unseasoned cement, but, as can be seen, cement under in-vitro conditions will clearly lose its strength properties. Preliminary comparison of the characteristics of the analysed CMW 1 and CMW 3 cements also reveals that CMW 1 usually achieved slightly worse strength parameters in all variants than CMW 3 cement samples in the same state of contamination and seasoning time, although statistical analysis may rule it out. The overall summary (Figure 3 and Figure 4) allows an attempt to read the correlations between the variable test parameters. Among other things, one can see a higher resistance of CMW 3 to loss of compressive strength with seasoning. The cured CMW 1 cement reaches lower values of average compressive strength much earlier, both in terms of the percentage increase in the degree of contamination and with a noticeably shorter seasoning time. The development of such a model may serve as a basis for consideration of the prediction of changes in the strength characteristics of cement along with further changes in the parameters analysed in the work. However, this seems to miss the objective, at least in terms of further increasing the contamination of cement, which in the presented work is large and even perhaps greater than the extent to which real, unconscious and unplanned contamination of cement is possible in operational practice (e.g., when cooling cement mass with saline).

As for the microhardness, apparently, the differences in the decrease of it over time, between cements in groups with the same degree of contamination, will not be statistically significant within these groups. However, there is a clear decrease in microhardness with an increase in contamination determined for the same seasoning time. Cements contaminated in the amount of 10% were characterized by a decrease in average strength in relation to uncontaminated ones by 15–20 Shore D, depending on the seasoning time. Analysing the two cements tested, it can be concluded that the trend line of the CMW 1 cement microhardness change is usually slightly less sloping than CMW 3, which would suggest its marginally higher resistance to contamination in terms of the investigated—surface microhardness feature. The presented summaries (Figure 7 and Figure 8), especially the contour diagram, show certain regularities when comparing both tested cements. In contrast to the compressive strength, for which CMW 1 was degraded faster than CMW 3, here CMW 3 proves less resistant to contamination and seasoning in saline solution. CMW 1 cement samples prepared under similar conditions and seasoned for an equal amount of time would usually have a higher average surface microhardness value than CMW 3. The nature of the changes in CMW 3 hardness is also clearly defined. As presented by the contour lines, it is the degree of contamination that directly influences the degradation of cement, especially at amounts >4% (at least in the test period of 30 days).

To summarize, the strength decrease has indeed been noted, as shown above in the result summary section, in particular in the results of statistical analysis. Interestingly, the decrease in compressive strength along with the degree of contamination between the least and most contaminated batches was twice as high for 30-day samples as for unseasoned ones. The unremarkable differences between the results of individual batches may suggest an exceedingly short time horizon of the test and exceedingly small intervals between measurements. It was also noted that the results of the samples made of CMW 1 cement were significantly less homogeneous in terms of the compressive strength values obtained than those made of CMW 3. However, this need not necessarily be due to specific differences between cements and, for example, the relatively small number of samples required for such tests by ISO 5833:2002 [41]. As observed in one of the batches, the heterogeneity of these results clearly stands out. Therefore, it is always worthwhile to make a few more samples, if only to be able to avoid clear outliers (incorrect samples) or measurement errors.

It was noted that the compressive strength of the samples containing 6 and 10%, respectively, of the impurities before seasoning did not meet the values set out in the above standard. For uncontaminated samples (0%), the strength dropped below the value specified in the standard only after 30 days of seasoning in saline solution.

What is important, in the presented studies, cement was contaminated in its entire volume, while in surgical practice the contamination may be more superficial. Nevertheless, even the surface degradation of the cement material can be fatal to the durability of the prosthesis anchorage in the bone and lead to loosening of the implant. In addition, development of a free space between the cement and the bone can provide optimal places for bacteria to colonise.

The fact that only static tests for compressive strength were performed should undoubtedly be included among the limitations of the presented tests, bearing in mind that in the conditions of actual operation, the cement is subjected to dynamic loads associated with every day, ordinary functioning of the patient. Another limitation was that test was performed on a standardized but relatively small number of samples within each of the tested batches. The next limitation concerned the in vivo environment and its temperature that was lower than the one in which the material works after implantation. Further work is planned to be carried out in the future, which will extend the works presented herein and will concern, among other things, the impact of seasoning conditions on the degree of degradation or fatigue testing of contaminated cements in conditions similar to actual working conditions.

## 5. Conclusions

The results of the tests presented in this study indicate that statistically significant deterioration of compression strength along with the amount of addition of physiological salt as a contaminant can be recorded already after 15 days, especially with a small amount of the above mentioned additive. The admixture of the analysed contaminant within the analysed range did not significantly affect the microhardness of the material. Degradation of this parameter of cement was recorded over the seasoning time, but mainly among samples with the degree of contamination higher than 6%.

## Figures and Tables

**Table 1:** Chemical composition of CMW 1 and CMW 3 bone cements.

Compound Name	CMW 1	CMW 3
**Bone Cement Powder Component:**		
Polymethyl methacrylate	88.85	83.88
Benzoyl peroxide	2.05	2.00
Barium sulphate	9.10	10.00
**Bone Cement Liquid Component:**		
Methyl methacrylate	98.50	97.5
*N*,*N*-dimethyl-p-toluidine	<2.00	<2.50
Hydroquinone (ppm)	75	75

**Table 2:** Average compressive strength values depending on degree of contamination and seasoning time.

Compression Strength [MPa]									
Saline Contamination	Seasoning Time >	CMW 1				CMW 3			
		0 Day	1 Day	15 Days	30 Days	0 Day	1 Day	15 Days	30 Days
0%	MV	89.23	78.85	72.07	70.26	85.45	85.15	76.02	70.38
	SD	5.23	7.37	7.29	6.88	2.79	3.50	8.62	3.54
	COV	5.9%	9.3%	10.1%	9.8%	3.3%	4.1%	11.3%	5.0%
1%	MV	85.35	86.63	71.65	68.78	89.01	80.68	88.91	72.67
	SD	8.30	7.76	7.50	13.37	6.87	3.67	6.16	6.84
	COV	9.7%	9.0%	10.5%	19.4%	7.7%	4.6%	6.9%	9.4%
3%	MV	74.46	77.38	72.97	65.20	76.67	74.50	76.68	71.22
	SD	3.83	4.65	2.58	3.98	2.67	2.41	4.56	3.20
	COV	5.1%	6.0%	3.5%	6.1%	3.5%	3.2%	5.9%	4.5%
6%	MV	67.96	69.57	66.48	63.46	72.89	70.95	71.86	65.91
	SD	4.29	5.20	5.30	5.71	8.14	3.89	2.28	3.38
	COV	6.3%	7.5%	8.0%	9.0%	11.2%	5.5%	3.2%	5.1%
10%	MV	63.89	69.11	66.37	57.50	65.50	66.52	64.02	63.41
	SD	3.92	7.42	5.29	2.80	4.07	3.23	3.55	4.39
	COV	6.1%	10.7%	8.0%	4.9%	6.2%	4.9%	5.5%	6.9%

MV—mean value of compression strength, SD—standard deviation of results and COV—coefficient of variation.

**Table 3:**
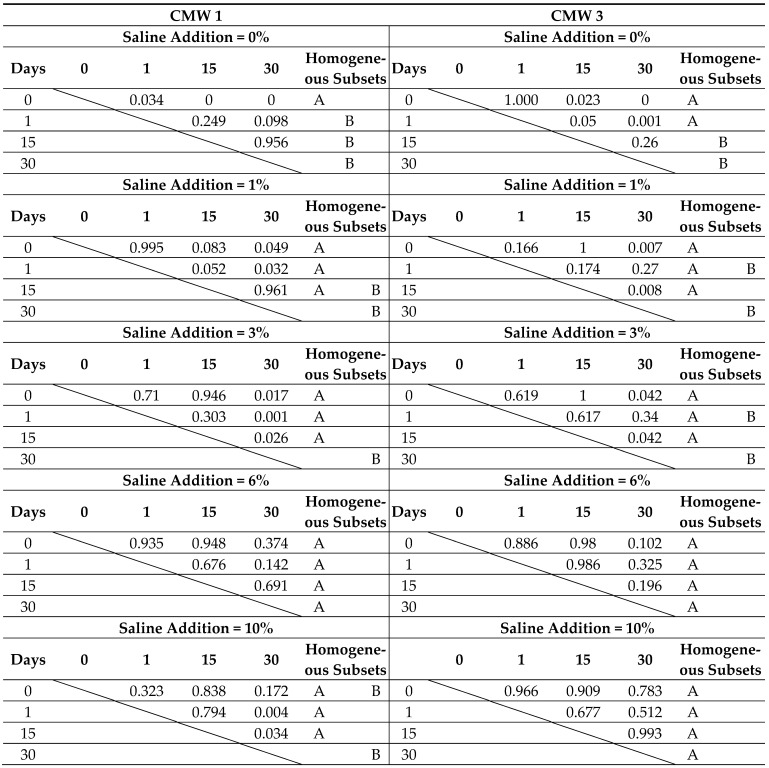
Summary of results of the statistical analysis of compressive strength test results depending on (**a**) seasoning time, grouped by degree of admixture.

**Table 4:**
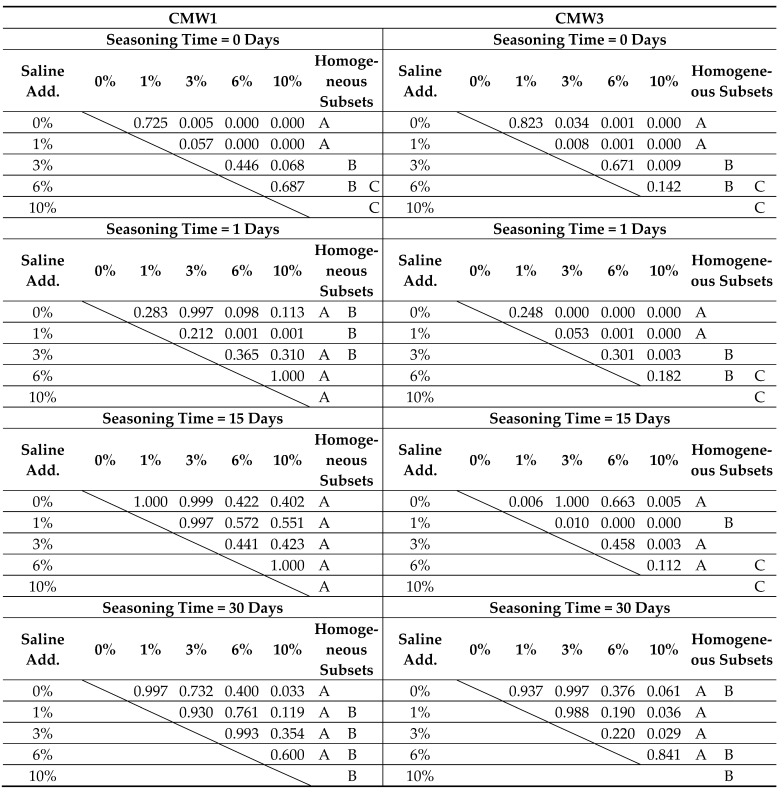
Summary of results of the statistical analysis of compressive strength test results depending on degree of admixture, grouped by seasoning time.

**Table 5:** Average microhardness values depending on degree of contamination and seasoning time.

Surface Microhardness [Shore D]									
Saline Contamination	Seasoning Time >	CMW1				CMW3			
		0 Days	1 Day	15 Days	30 Days	0 Days	1 Day	15 Days	30 Days
0%	MV	73.09	74.21	72.18	71.46	80.00	77.19	77.13	74.41
	SD	4.92	5.00	5.88	4.45	3.72	2.47	2.33	1.71
	COV	6.7%	6.7%	8.1%	6.2%	4.7%	3.2%	3.0%	2.3%
1%	MV	74.30	76.05	76.67	73.93	78.77	75.53	76.83	72.79
	SD	3.86	4.48	3.85	6.89	3.16	2.88	5.94	5.00
	COV	5.2%	5.9%	5.0%	9.3%	4.0%	3.8%	7.7%	6.9%
3%	MV	75.97	73.24	73.45	70.50	80.14	71.01	71.01	71.74
	SD	3.84	5.64	2.90	4.48	5.12	3.80	8.14	3.60
	COV	5.0%	7.7%	3.9%	6.4%	6.4%	5.4%	11.5%	5.0%
6%	MV	76.14	71.17	74.87	68.48	75.22	70.59	67.21	67.69
	SD	3.57	2.70	4.07	6.93	3.60	3.03	4.48	3.95
	COV	4.7%	3.8%	5.4%	10.1%	4.8%	4.3%	6.7%	5.8%
10%	MV	68.25	64.65	66.24	63.05	65.84	60.17	58.25	59.29
	SD	7.44	3.85	5.67	6.78	3.19	4.80	2.84	4.69
	COV	10.9%	6.0%	8.6%	10.8%	4.9%	8.0%	4.9%	7.9%

MV—mean value of microhardness in Shore D scale, SD—standard deviation of results and COV—coefficient of variation.

**Table 6:** Summary of results of the statistical analysis of microhardness test results depending on seasoning time, grouped by degree of admixture.

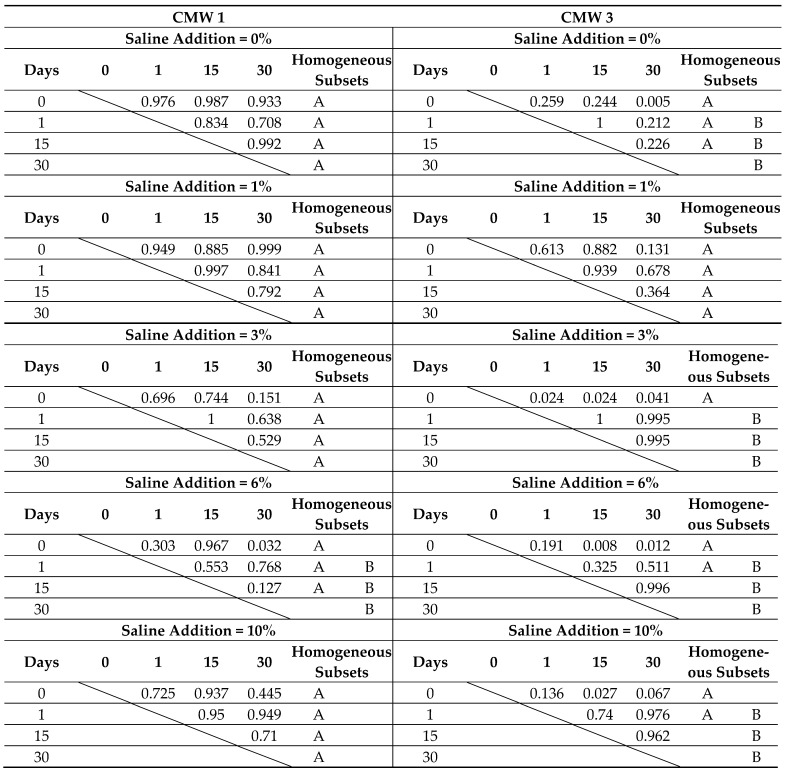

**Table 7:** Summary of results of the statistical analysis of microhardness test results depending on degree of admixture, grouped by seasoning time.

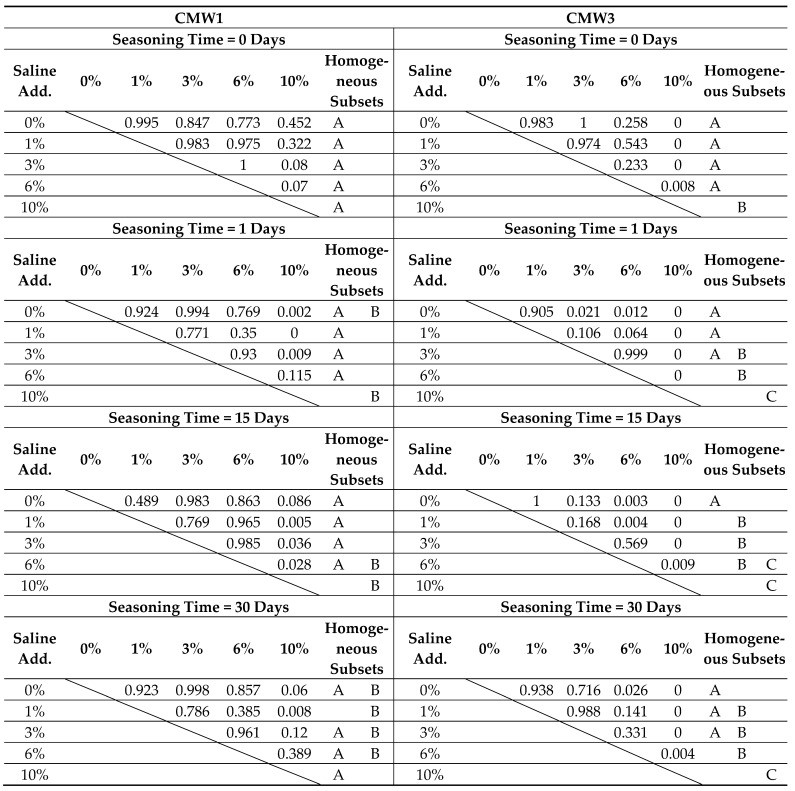

## Data Availability

The data presented in this study are available on request from the corresponding authors.
